# Pro-epileptogenic effects of viral-like inflammation in both mature and immature brains

**DOI:** 10.1186/s12974-016-0773-6

**Published:** 2016-12-12

**Authors:** Nina Dupuis, Andrey Mazarati, Béatrice Desnous, Vibol Chhor, Bobbi Fleiss, Tifenn Le Charpentier, Sophie Lebon, Zsolt Csaba, Pierre Gressens, Pascal Dournaud, Stéphane Auvin

**Affiliations:** 1 INSERM, U1141, 75019 Paris, France; 2 Université Paris Diderot, Sorbonne Paris Cité, INSERM UMR1141, 75019 Paris, France; 3 Department of Pediatrics, Neurology division and Children’s Discovery and Innovation Institute, David Geffen School of Medicine at UCLA, Los Angeles, CA 90095 USA; 4 AP-HP, Hôpital Robert Debré, Service de Neurologie Pédiatrique, 75019 Paris, France

**Keywords:** Encephalitis, Epileptogenesis, Kindling, Microglia, Minocycline, Toll-like receptor, Virus

## Abstract

**Background:**

Infectious encephalitides are most often associated with acute seizures during the infection period and are risk factors for the development of epilepsy at later times. Mechanisms of viral encephalitis-induced epileptogenesis are poorly understood. Here, we evaluated the contribution of viral encephalitis-associated inflammation to ictogenesis and epileptogenesis using a rapid kindling protocol in rats. In addition, we examined whether minocycline can improve outcomes of viral-like brain inflammation.

**Methods:**

To produce viral-like inflammation, polyinosinic-polycytidylic acid (PIC), a toll-like receptor 3 (TLR3) agonist, was applied to microglial/macrophage cell cultures and to the hippocampus of postnatal day 13 (P13) and postnatal day 74 (P74) rats. Cell cultures permit the examination of the inflammation induced by PIC, while the in vivo setting better suits the analysis of cytokine production and the effects of inflammation on epileptogenesis. Minocycline (50 mg/kg) was injected intraperitoneally for 3 consecutive days prior to the kindling procedure to evaluate its effects on inflammation and epileptogenesis.

**Results:**

PIC injection facilitated kindling epileptogenesis, which was evident as an increase in the number of full limbic seizures at both ages. Furthermore, in P14 rats, we observed a faster seizure onset and prolonged retention of the kindling state. PIC administration also led to an increase in interleukin 1β (IL-1β) levels in the hippocampus in P14 and P75 rats. Treatment with minocycline reversed neither the pro-epileptogenic effects of PIC nor the increase of IL-1β in the hippocampus in both P14 and P75 rats.

**Conclusions:**

Hippocampal injection of PIC facilitates rapid kindling epileptogenesis at both P14 and P75, suggesting that viral–induced inflammation increases epileptogenesis irrespective of brain maturation. Minocycline, however, was unable to reverse the increase of epileptogenesis, which might be linked to its absence of effect on hippocampal IL-1β levels at both ages.

**Electronic supplementary material:**

The online version of this article (doi:10.1186/s12974-016-0773-6) contains supplementary material, which is available to authorized users.

## Background

Infectious encephalitides are most often associated with seizures during the infection period [1, 2] and are established risk factors for the development of epilepsy at later times [1, 3, 4]. Although many infectious encephalitides are of unknown origin, the majority of clinical studies suggest a viral etiology [5, 6]. Common viruses associated with encephalitis-induced seizures are herpes simplex and cytomegalovirus. Children are three times more prone to viral encephalitis than adults are [6, 7], moreover, infants under 1 year are particularly affected [6]. Mechanisms leading to increased seizure occurrence at the time of infection and those underlying encephalitis-induced epileptogenesis are poorly understood. Virus-induced cell injury, brain inflammation, and initial prolonged seizures appear to be key contributors to epileptogenesis after the acute phase of encephalitis [2, 8–10].

There are several models of virus-induced encephalitis. However, most experimental animals die during the acute infection period, rendering the study of epileptogenesis impossible [11]. The use of the Daniel’s (DA) strain of Theiler’s murine encephalomyelitis virus (TMEV) in C57Bl6 mice leads to behavioral seizures during the acute phase in 75% of the animals [10]. A significant proportion (65%) of the animals with acute symptomatic seizures develops spontaneous recurrent seizures later in life [10]. In this model, macrophages have been shown to be associated with seizures during the acute phase of encephalitis, while neuronal loss and inflammation contribute to epileptogenesis [8, 12].

In the central nervous system (CNS), the innate inflammatory response to viral infection primarily involves the activation of microglia, which are cerebral monocyte-like immune cells [13]. The innate inflammatory response is triggered by pattern-recognition receptors, including toll-like receptors (TLRs), RIG1-like receptors, and nucleotide-binding oligomerization domain-like receptors [14]. TLR3 is a receptor for double-stranded RNA, which is released by viruses and necrotic cells. Upon activation, TLR3 triggers cytokine and interferon production pathways [15, 16].

Clinical and experimental data suggest an important role for neuroinflammation in seizure occurrence and epileptogenesis [17]. A direct role of cytokines, and particularly of interleukin 1β (IL-1β), has been demonstrated in the precipitation of seizures during brain inflammation. Cytokines have also been implicated in epileptogenesis. Therapeutic targeting of IL-1β has been proven effective in inhibiting epileptogenesis [18, 19].

Since encephalitis in humans is more frequent in children than in adults, we decided to evaluate the contribution of brain viral-like inflammation to epileptogenesis in not only mature but also in immature brains. To induce brain viral-like inflammation, we injected a TLR3 agonist, polyinosinic-polycytidylic acid (PIC) [20], into the hippocampus, which produces an inflammatory response in the absence of viral replication. Unlike the TMEV model, this model allows us to explore the role of brain inflammation in the absence of viral replication-induced brain injury. Moreover, our kindling model, which is a model of compressed epileptogenesis, allows us to perform the experiment during a precise window of brain maturation.

We first characterized the response to PIC both in vitro and in vivo. Since macrophages contribute to epileptogenesis in the TMEV model, we decided to study microglial cells and macrophages to evaluate potential differences that might derive from specific contributions of these cell types. The second part of the study consisted of the assessment of the effects of intrahippocampal PIC injection on hippocampal excitability and epileptogenesis using the rapid kindling model. We conducted the study in P14 and P75 animals to evaluate whether brain maturation contributes to a higher susceptibility to inflammatory responses and epileptogenesis. Finally, we evaluated the effects of minocycline, an antibiotic described as a modulator of inflammation [21–23], on the inflammatory response to and the modulation of seizures induced by PIC.

## Methods

### Animals

Male Wistar rats (Charles River, L’Arbesle, France) were used for the rapid kindling and cytokine-dosage experiments. We used P13 and P74 rats at the time of the PIC intrahippocampal injection. Primary microglial and macrophage cell cultures were prepared from the neocortex of OF1 mice (Charles River, L’Arbesle) and 2-month-old OF1 male mice (Charles River). Animals were housed in standard laboratory conditions with controlled temperature/humidity, a 12:12-hour light/dark cycle, and free access to food and water. Studies were approved by the animal ethical institutional review committee (Bichat-Robert Debré ethical committee, Paris, France, #2015072801547679) and met stipulations of the guide for the care and use of laboratory animals (NIH, Bethesda, Maryland, USA), as well as recommendations of reduction, refinement, and replacement (known as the 3 Rs) [24].

### Drugs

PIC and minocycline (Sigma, Lyon, France) were dissolved in saline for the in vivo experiments and in phosphate buffered saline (PBS) for the in vitro experiments. PIC (10 μg/rat) was injected stereotaxically into the ventral hippocampus 24 h before rapid kindling at the following coordinates: P13, 3.0 mm posterior, 3.9 mm left, 4.2 mm ventral; P74, 4.8 mm posterior, 5.3 mm left, 6.5 mm ventral, in relation to bregma. The injection of PIC into the hippocampus aims to mimic encephalitis, which is characterized by brain inflammation, rather than meningitis, which is inflammation in the meninges and the cerebrospinal fluid (CSF). Minocycline (50 mg/kg) was administered as 3 consecutive intraperitoneal (i.p.) injections 48, 24, and 4 h before the start of the kindling protocol. Table 1 summarizes the in vitro and in vivo experiments.

**Table 1:** Experimental plan summarizing treatments and experiment (exp.) numbers included in the study

Cell culture experiments	In vivo experiments
Microglia	Rapid kindling protocol
RT-PCR: PBS vs. PIC, *n* = 5 (x3 exp.)	P14: saline (*n* = 5) vs. PIC (*n* = 6)
Luminex: PBS vs. PIC, *n* = 5 (x1 exp.)	P75: saline (*n* = 6) vs. PIC (*n* = 7)
RT-PCR: PIC vs. PIC + Mino, *n* = 5 (x3 exp.)	P14: mino (*n* = 7) vs. PIC + Mino (*n* = 7)
Luminex: PIC vs. PIC + Mino, *n* = (x1 exp.)	P75: mino (*n* = 5) vs. PIC + Mino (*n* = 5)
Macrophages	Cytokines dosage by luminex
RT-PCR: PBS vs. PIC, *n* = 5 (x3 exp.)	P14: saline i.p. + saline i.h. (*n* = 5)
Luminex: PBS vs. PIC, *n* = 5 (x1 exp.)	Saline i.p. + PIC i.h. (*n* = 5)
RT-PCR: PIC vs. PIC + mino, *n* = 5 (x3 exp.)	Mino i.p. + saline i.h. (*n* = 5)
Luminex: PIC vs. PIC + mino, *n* = 5 (x1 exp.)	Mino i.p. + PIC i.h. (*n* = 5)
	P75: Saline i.p. + saline i.h. (*n* = 5)
	Saline i.p. + PIC i.h. (*n* = 5)
	Mino i.p. + saline i.h. (*n* = 5)
	Mino i.p. + PIC i.h. (*n* = 5)

### Microglia primary cell cultures

Primary mixed glial cell cultures were prepared from the neocortices of P0-P1 OF1 mice, as described previously [25, 26]. Briefly, the cortices were dissected and the meninges were removed in 0.1 M PBS with 6% glucose and 2% penicillin–streptomycin (PS, Gibco, Cergy Pontoise, France). The tissue was subsequently mechanically dissociated and spun at 1000 rpm for 5 min at 4 °C. The pellet was resuspended in low-glucose Dulbecco’s modified Eagle’s minimum essential medium (DMEM, 31885, Gibco) supplemented with 10% fetal bovine serum (FBS, Gibco) and 0.01% PS. The cells were then plated in poly-D-L-ornithine-coated T75 flasks. Microglia were isolated from the primary mixed glial cultures on day in vitro 14 by shaking for 20 min. The supernatant was collated and spun at 1800 rpm for 5 min at 4 °C. Microglia were resuspended in DMEM supplemented with 0% FBS in 6-well culture plates and cultured for 1 day before treatment.

Microglia were exposed for 4 h to PBS or PIC (1 μg/ml). Supernatants were collected and stored at −80 °C until cytokine levels were measured. Cells were harvested and RNA was extracted for gene expression analysis.

### Peritoneal macrophage primary cell cultures

Primary peritoneal macrophage cultures were prepared from 2-month-old OF1 male mice, as described previously [27]. Macrophages were isolated by peritoneal lavage with ice-cold PBS. After 400 × *g* centrifugation, cells were resuspended in DMEM/F12 medium (Gibco, Cergy Pontoise, France) supplemented with 10% FBS (Gibco) and 0.01% PS (Gibco) in 6-well culture plates. After 1 h, non-adherent cells were removed by washing and adherent cells were found to be ~95% pure based on morphological criteria. Cells were cultured for 1 day before treatment.

Similar to microglia, macrophages were exposed to PBS or PIC (4-hour; 1 μg/ml). Supernatants were collected and stored at −80 °C until cytokine level measurements. Cells were harvested and RNA was extracted for gene expression analysis.

### RNA extraction and quantitative PCR

Total RNA from primary microglial cell cultures was extracted using the RNeasy mini kit according to the manufacturer’s instructions (Qiagen, Courtaboeuf, France). Total RNA (500 ng) was subjected to reverse transcription based on equal amounts of RNA using the iScript™ cDNA synthesis kit (Bio-Rad, Marnes-la-Coquette, France). Quantitative PCR was then performed in duplicate for each sample using the SYBR Green Supermix (Bio-Rad) for 40 cycles with a two-step program (5 s of denaturation at 96 °C, and 10 s of annealing at 60 °C). The primers used are summarized in Table 2. The relative expression of genes of interest was compared with that of the reference gene, glyceraldehyde-3-phosphate dehydrogenase (Gapdh). Analyses were performed using Biorad CFX Manager 3.0 software.

**Table 2:** List of PCR primers used in the study

Gene	Forward primer (5′−3′)	Reverse primer (5′−3′)
Gapdh	GGC CTT CCG TGT TCC TAC	TGT CAT CAT ACT TGG CAG GTT
Il1β	GGG CCT CAA AGG AAA GAA TC	TCT TCT TTG GGT ATT GCT TGG
Il6	CAA AGC CAG AGT CCT TCA GA	GCC ACT CCT TCT GTG ACT CC
Tnfα	GCC TCT TCT CAT TCC TGC TT	AGG GTC TGG GCC ATA GAA CT
Il1rn	TTG TGC CAA GTC TGG AGA TG	TTC TCA GAG CGG ATG AAG GT
NfκB	TTA CAT TCC ATC CCG GAG TC	GCA CAA TCT TTA GGG CCA TT
IκB	CTC ACG GAG GAC GGA GAC T	GTC TCC CTT CAC CTG ACC AA
Ifnβ	TGA ACT CCA CCA GCA GAC AG	GGA CAT CTC CCA CGT CAA TC
Trif	GCT CCA GGC TTC ATT CTC C	AAG GCA CCT AGA ATG CCA AA
Tbk1	ATA AGC TTC CTT CGC CCA GT	CCA CAG GGA CAA AAC TCC AT
Irf3	GAT GGC TGA CTT TGG CAT CT	GAC ACG TCC GGC TTA TCC T
Ptgs2	TCA TTC ACC AGA CAG ATT GCT	AAG CGT TTG CGG TAC TCA TT
Nos2	CCC TTC AAT GGT TGG TAC ATG G	ACA TTG ATC TCC GTG ACA GCC

### Multiplex cytokine assay

Freshly excised hippocampi from P14 and P75 rats (24 h after ventral hippocampal injection) were homogenized and total protein was extracted in PBS supplemented with protease inhibitors (Roche Diagnostics, Meylan, France). After a 12500-rpm centrifugation for 30 min, supernatants were collected.

IL-1β, interleukin 6 (IL-6), tumor necrosis factor α (TNFα), and interleukin 10 (IL-10) levels were measured in microglia supernatants and hippocampal protein extracts using a Bio-plex 200 and a 96-well magnetic plate assay according to the manufacturer’s instructions (Biorad Laboratories, Marnes la Coquette, France). All samples were run in duplicate, and data were analyzed using Bio-Plex Manager software. For hippocampal measurements, cytokine levels were expressed relative to total protein levels (pg of cytokine/mg of total protein).

### Immunohistochemistry

Twenty-four hours after the hippocampal PIC injection, P14 and P75 rats (*n* = 5 for each experimental group) underwent transcardiac perfusion with 4% paraformaldehyde. Coronal 30-μm-thick free-floating sections were immunolabeled overnight with rabbit polyclonal anti-ionized calcium binding adaptor molecule 1 (Iba1) (1:1000, Wako) and mouse monoclonal anti-glial fibrillary acidic protein (GFAP) (1:500, Sigma) antibodies and revealed by Alexa Fluor 488- and Cy3-conjugated anti-rabbit and anti-mouse secondary antibodies (1:500, Invitrogen).

Sections of the ventral hippocampus at the PIC or saline injection sites were imaged using a fluorescence microscope (Zeiss Axio Observer Z1). Immunopositive cells were quantified by a cell count in six different areas distant from the injection site in each animal (square; Fig. 3) using ImageJ software.

### Rapid kindling protocol

Animals were anesthetized with isoflurane, stereotaxically injected with 10 μg of PIC (1 μl in the left hippocampus), and implanted with a bipolar stimulating electrode (Plastics One Inc., Roanoke, VA) into the left ventral hippocampus. The coordinates in relation to bregma were as follows: P13, 3.0 mm posterior, 3.9 mm left, 4.2 mm ventral; P74, 4.8 mm posterior, 5.3 mm left, 6.5 mm ventral. A tripolar recording electrode was wrapped around the skull screws above the right hemisphere. Electrodes were fixed to the skull using Integrity composite resin (Densply, York, PA, U.S.A.).

Twenty-four hours after surgery, the animals were connected to a DS8000 electrical stimulator via DLS100 stimulus isolators (World Precision Instruments, Sarasota, FL, U.S.A.). Electroencephalograms (EEGs) were acquired using the MP100/EEG100B acquisition system and AcqKnowledge software (BIOPAC, Santa Barbara, CA, U.S.A.). The electrical stimulus used for the assessment of the ictogenesis parameters and the kindling protocol was a square wave biphasic electrical stimulus with the following characteristics: 10-second train duration, 20 Hz, 1-ms pulse duration, delivered every 5 min. At 24 and 48 h post-injection, afterdischarge threshold (ADT) and afterdischarge duration (ADD) were assessed using 0.1 mA incremental currents delivered every 5 min. An AD was defined as paroxysmal epileptiform activity lasting at least 5 s with peak-to-peak amplitude of at least twice the baseline background EEG observed after the end of the hippocampal electrical stimulation.

The kindling protocol consisted of 60 trains delivered every 5 min with a current of 0.1 mA over the ADT with parameters otherwise similar to those described for afterdischarge detection. Animals were video-recorded during the kindling procedure. Behavioral seizures were scored using Racine’s scale: 1, motor arrest and twitching vibrissae; 2, chewing, head bobbing; 3, forelimb clonus; 4, forelimb clonus and rearing; 5, rearing and falling. Epileptogenesis was analyzed by calculating the numbers of kindling trials needed to first reach a stage 4–5 seizure and the total numbers of stage 4–5 seizures during the kindling procedure. Ictogenesis was analyzed by calculating changes in afterdischarge properties 24 h after the kindling procedure compared to baseline afterdischarge properties. We also determined the severities of behavioral seizures in response to the threshold stimulation in the kindled animals [28–30].

### Statistical analysis

Data were analyzed using Prism 5 software (Graphpad, San Diego, CA, U.S.A.). Data were expressed as mean ± standard errors of the mean. Statistical analyses were performed using Kruskal-Wallis followed by Dunn’s posthoc tests and the Mann-Whitney test to compare two groups.

## Results

### PIC triggers inflammatory responses in both microglia and macrophages

**Fig. 1:**
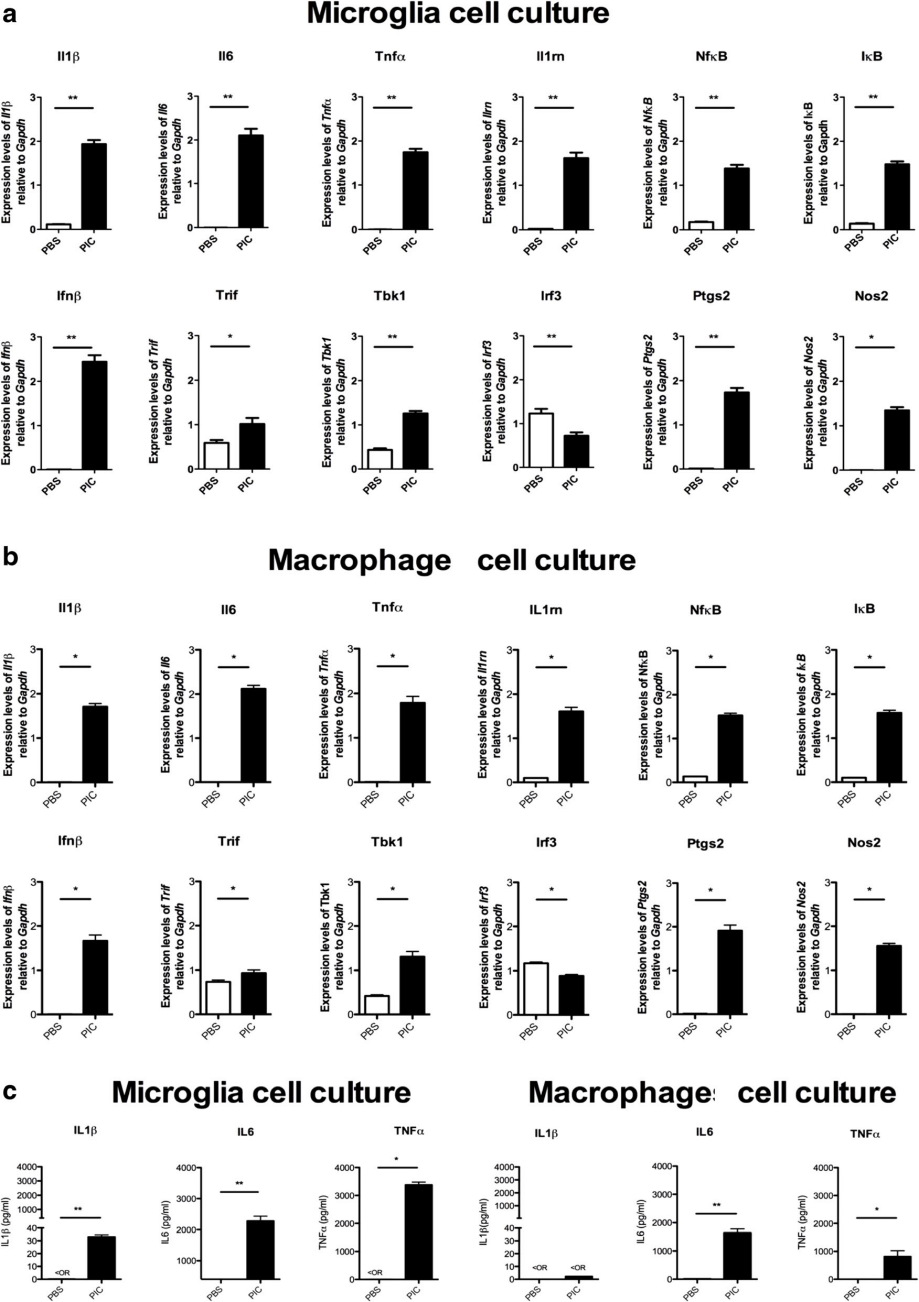
Inflammatory response to PIC treatment in microglial and macrophage primary cell cultures. mRNA quantification by RT-qPCR of inflammatory actors in microglial (**a**) and macrophage (**b**) primary cultures 4 h after PBS or PIC exposure (1 μg/ml). Cytokine profile (IL1β, IL6, TNFα) 4 h after PBS or PIC exposure (1 μg/ml) in primary microglial and macrophage cell culture supernatants (**c**). Data are presented as mean ± SEM. *p < 0.05, **p < 0.01 vs. PBS (Mann-Whitney test)

We produced primary cell cultures of both microglia and macrophages to study inflammatory responses to PIC. In microglial cells, PIC treatment increased mRNA levels of Il-1β, Il-6, Tnfα, interferon β (Infβ*)*, and interleukin 1 receptor antagonist (IL-1rn), as well as of activators of TLR3 transduction pathways (i.e., nuclear factor kappa-light-chain-enhancer of activated B cells (NfκB), nuclear factor of kappa light polypeptide gene enhancer in B-cells inhibitor, alpha (IκB), TIR-domain-containing adapter-inducing interferon-β (Trif), and TANK-binding kinase (Tbk1)). Ptgs2 and Nos2 (coding for cyclooxygenase2 and inducible nitric oxide synthase proteins, respectively) mRNA levels were also increased after PIC exposure (Fig. 1a). The levels of pro-inflammatory cytokines were consistent with the reverse transcriptase-PCR findings and indicated increases in the levels of IL-1β, IL-6, and TNFα after PIC exposure (Fig. 1c). In macrophages, we observed a similar profile of inflammatory activation by studying mRNA expression after PIC exposure (Fig. 1b). We found increases of IL-6 and TNFα not of IL-1β in the supernatant after PIC exposure (Fig. 1c).

### Hippocampal injection of PIC triggers an inflammatory IL-1β response

**Fig. 2:**
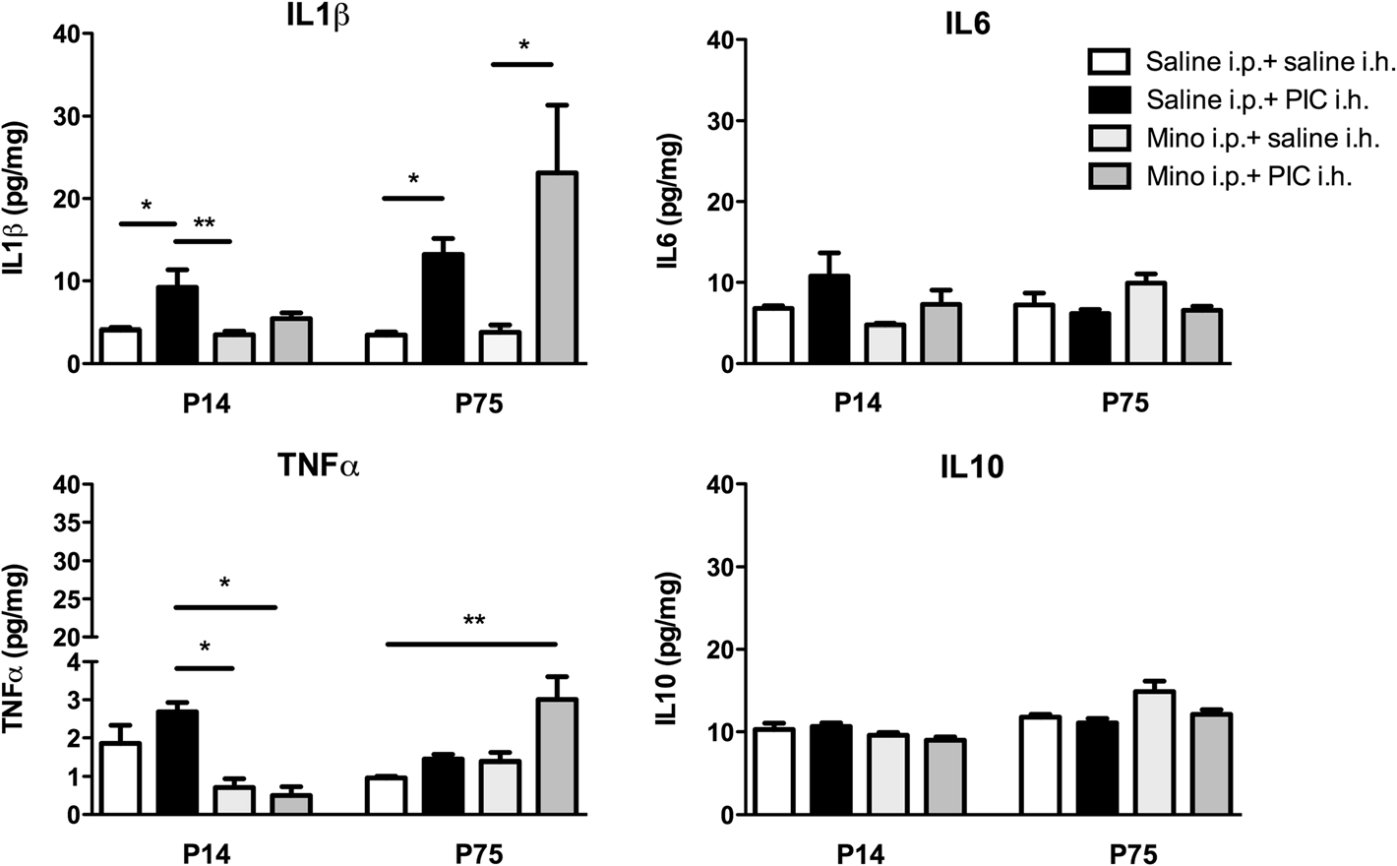
Hippocampal cytokine levels 24 h after PIC injection. Cytokine profiles (IL1β, IL6, IL10, TNFα) in the hippocampus 24 h after intrahippocampal injection (saline i.p. + saline i.h., saline i.p. + PIC i.h. (10 μg/rat), minocycline i.p. (50 mg/kg) + saline i.h. or minocycline i.p. (50 mg/kg) + PIC i.h. (10 μg/rat)) in P14 and in P75 rats. Data are presented as mean ± SEM. *p < 0.05 vs. all other treatment groups according to age group (Kruskal-Wallis test)

Following the injection of PIC into the hippocampus, we observed an increase in IL-1β compared to the controls at both ages (Fig. 2). In contrast, IL-6, TNFα and IL-10 protein levels were unchanged 24 h after PIC injection at both ages (Fig. 2). The intracerebral PIC injection did not result in any change in the blood cytokine levels (Additional file 1).

### Hippocampal injection of PIC does not change the numbers of glial cells in vivo

**Fig. 3:**
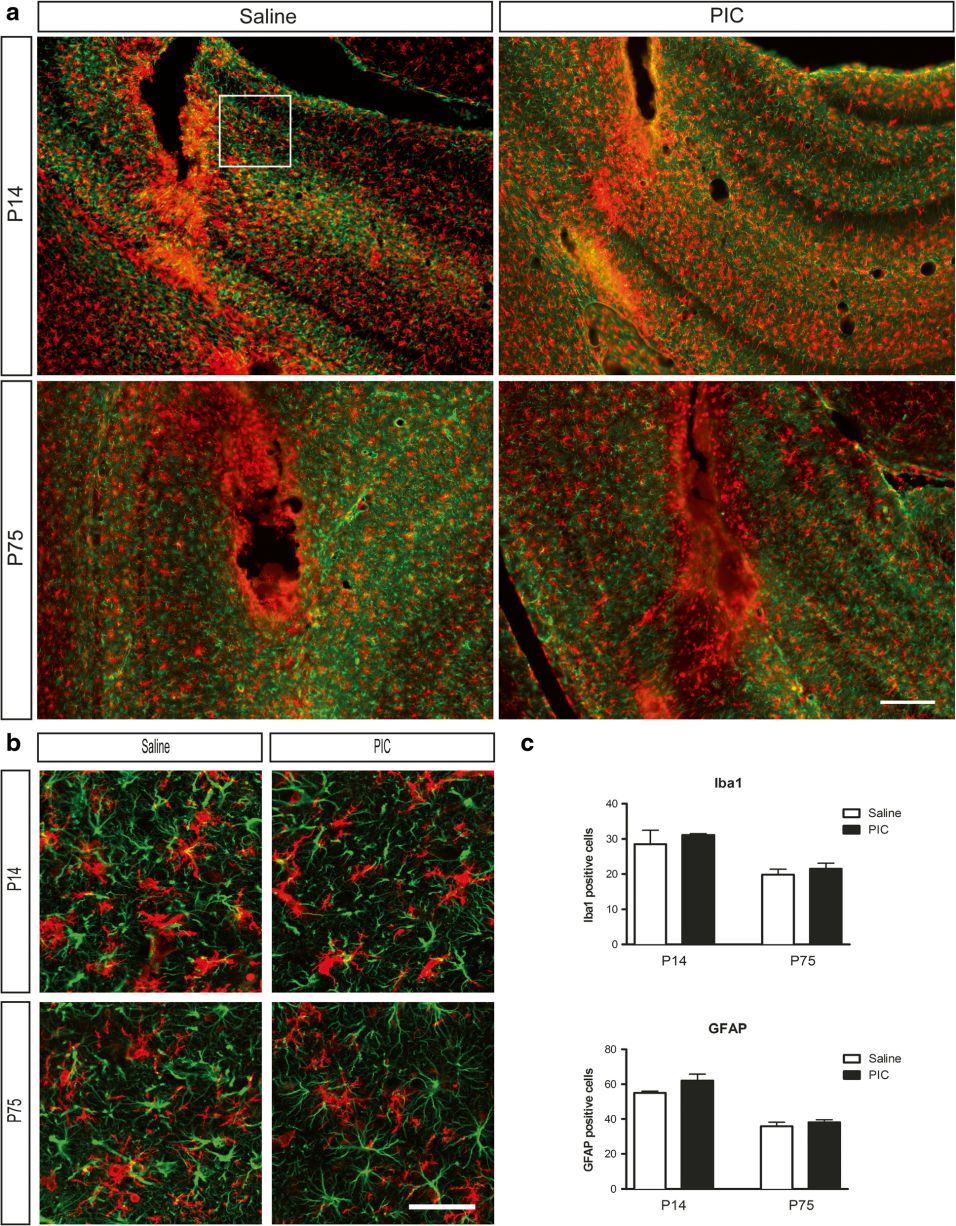
Glial activation at the injection site 24 h after PIC or saline injection. Low (**a**) and high (**b**) magnifications of representative pictures of microglial cells (Iba1, *red*) and astrocytes (GFAP, *green*) in the hippocampus 24 h after intrahippocampal injection (saline vs. PIC 10 μg/rat) in P14 and P75 rats. The *white rectangle* represents an area of cell count. Quantifications of microglial cell (Iba1) and astrocyte (GFAP) number (**c**). Data are presented as mean ± SEM. *p < 0.05, **p < 0.01, ***p < 0.001 vs. saline (Mann-Whitney test). *Scale bars*: **a** 200 μm; **b** 50 μm

In order to determine whether IL-1β production is associated with glial activation, we analyzed microglia and astrocyte distribution in the hippocampus. Twenty-four hours after the intrahippocampal injection of PIC, the density of Iba1-expressing cells was not different from those found in controls in either age group (Fig. 3). Similarly, in both age groups, the density of GFAP-expressing cells was not different from that of the controls (Fig. 3). Around the injection site, where we observed glial activation (Fig. 3), no difference in the relative intensities of both Iba1- and GFAP immunoreactive signals was observed in PIC-injected vs. saline control animals (Fig. 3c).

### Hippocampal injection of PIC facilitates epileptogenesis in both P14 and P75 rats

**Fig. 4:**
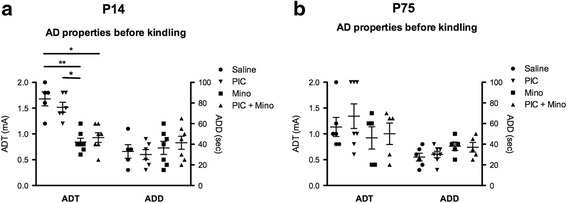
Effect of PIC and minocycline treatments on hippocampal excitability in P14 and P75 rats. Afterdischarge threshold (ADT) and afterdischarge duration (ADD) 24 h after intrahippocampal injection (saline vs. PIC 10 μg/rat) and the last minocycline intraperitoneal injection (Saline + minocycline vs*.* PIC 10 μg/rat + minocycline) in P14 (**a**) and in P75 rats (**b**). Data are presented as mean ± SEM. **p < 0.01 vs. saline (Mann-Whitney test)

We used a rapid kindling model to determine whether intrahippocampal injection of PIC produces pro-ictogenic and/or pro-epileptogenic effects. First, AD parameters were not changed by PIC administration. This suggests that there were no changes in ambient hippocampal excitability at either P14 or P75 (Fig. 4a, b).

**Fig. 5:**
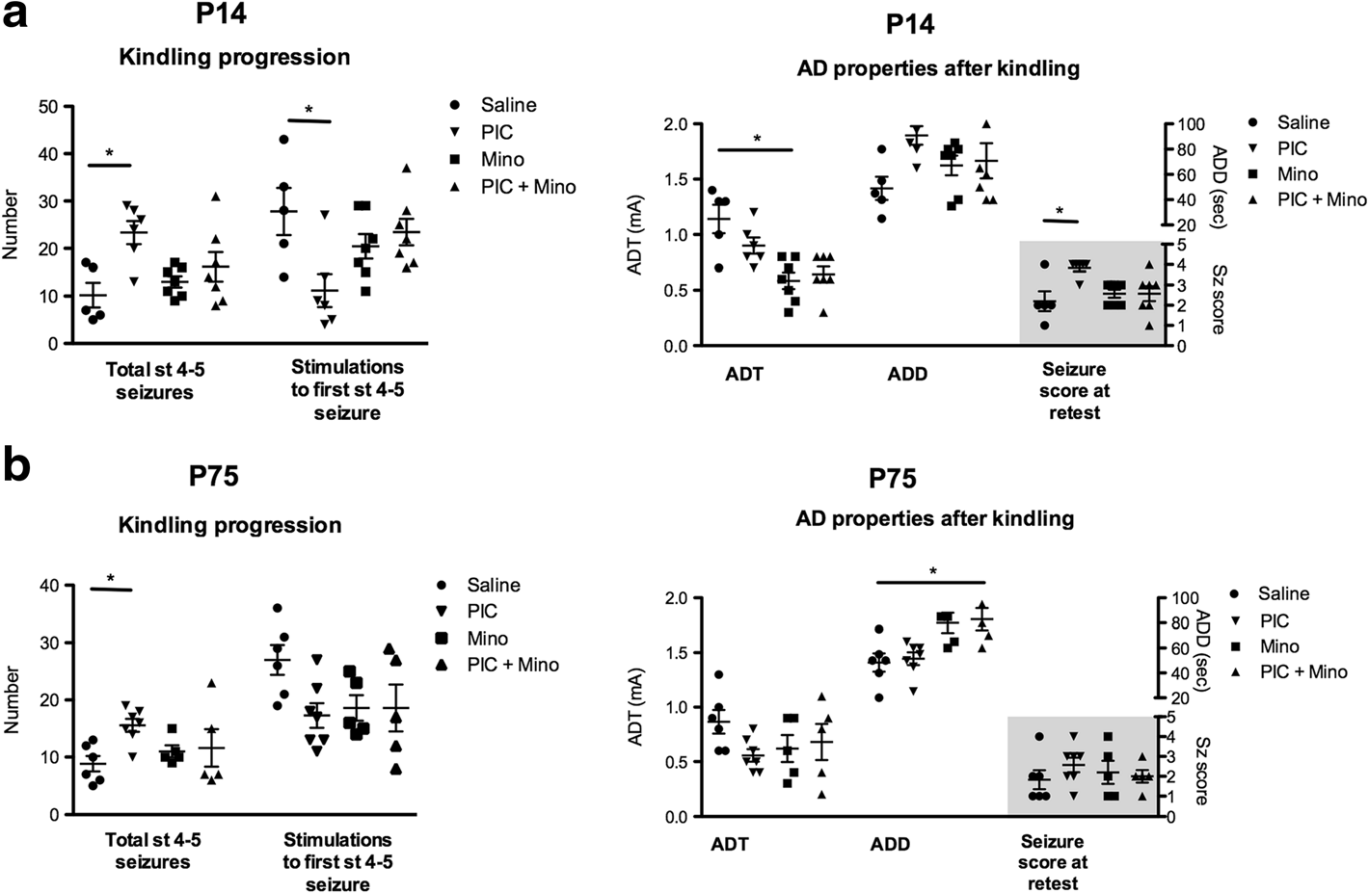
Effects of PIC and minocycline treatments on epileptogenesis. Kindling progression parameters 24 h after intrahippocampal injection (saline vs. PIC 10 μg/rat) and the last minocycline intraperitoneal injection (Saline + minocycline vs. PIC 10 μg/rat + minocycline) and afterdischarge (AD) properties at retest, 24 h after kindling onset in P14 (**a**) and in P75 rats (**b**). Total number of stages 4-5 seizures and number of stimulations to achieve stage 4–5 seizure in P14 (**a**) and P75 rats (**b**). Afterdischarge threshold (ADT), afterdischarge duration (ADD), and seizure score at retest in P14 (**a**) and P75 rats (**b**) 24 h after kindling onset. Data are presented as mean ± SEM. *p < 0.05 vs. saline (Mann-Whitney test)

Second, kindling epileptogenesis was facilitated by PIC; this was evident as an increase in the numbers of full limbic (i.e., stage 4–5) seizures (P14: 23.3 ± 2.4 vs. 10.2 ± 2.6, *p* < 0.01; P75: 15.6 ± 1.1 vs. 8.8 ± 1.3, *p* < 0.01; comparisons are for PIC vs. saline) and a faster onset of stage 4–5 seizures in P14 animals (number of trials to first stage 4–5 seizure: P14, 11.2 ± 3.5 vs*.* 27.8 ± 5.0, *p* < 0.05; comparison is for PIC vs. saline) (Fig. 5a, b).

Third, the modification of AD properties after kindling by PIC was more evident in P14 rats. 24 h after the kindling procedure, PIC further increased the scores of seizures induced by threshold stimulation in P14 rats (Fig. 5a). There was no difference in AD threshold between the PIC and control groups at P14 (Fig. 5a). None of these parameters (AD parameters and seizure scores at retest) were changed in P75 rats (Fig. 5b).

### Inefficiency of minocycline in the PIC-facilitated epileptogenesis in both P14 and P75 rats

To test possible modulations of the pro-epileptogenic effects of PIC by minocycline, we administered minocycline (50 mg/kg i.p.) over three consecutive days to animals at both ages, with and without intrahippocampal PIC injections (Figs. 4 and 5). First, in AD parameters, we detected a decrease of ADT in minocycline-treated P14 animals as compared to saline controls (Fig. 4a). Second, kindling epileptogenesis facilitated by PIC was not inhibited by minocycline (Fig. 5). Third, after kindling completion, minocycline did not reduce the pro-epileptogenic effects of PIC (Fig. 5). On the contrary, the ADT in minocycline-treated P14 animals was still decreased as compared to saline controls (Fig. 5a).

### Minocycline does not modulate IL-1β level in the hippocampus

Hippocampal level of IL-1β increased by PIC was not modified by minocycline treatment at both ages. TNFα was however reduced in the PIC-injected group by minocycline in P14 rats as compared to controls (Fig. 2).

## Discussion

In this study, we demonstrated that PIC induced an inflammatory response in both microglial cells and macrophages in vitro*.* It also led to a pro-inflammatory response in the hippocampi of both P14 and P75 rats but was limited to an increase of IL-1β. Furthermore, PIC accelerated epileptogenesis at both ages without changing baseline hippocampal excitability. Using minocycline as an anti-inflammatory agent, we were not able to reverse the pro-epileptogenic effects of PIC. This might be attributed to its inability to change the IL-1β hippocampal levels in our experimental settings.

PIC mimics viral infection via binding to TLR3 [15, 16, 20] and results in an inflammatory response [31]. However, there are limited data regarding the exact role of microglial cells in the viral-like inflammatory response induced by PIC [20]. In our primary microglial cultures, PIC resulted in the activation of TLR3 downstream pathways (i.e., increased mRNA expression levels of Trif, NfκB/IκB, and Tbk1) and in the release of IL-1β, IL-6, and TNFα proteins. This is consistent with previous work using higher doses of PIC (50 μg/ml) [20]. We found a similar inflammatory response in both microglia and macrophages, the only difference being that IL-1β was significantly increased by PIC only in microglial cell cultures. This might be a characteristic of our model, as IL-6 release by both infiltrating macrophages and resident microglia has been observed in previous virus-induced encephalitis models, and has been shown to contribute to the precipitation of seizures [8, 9, 32].

The injection of PIC into the hippocampus indeed resulted in an increase in IL-1β levels in both age groups. This was not associated with any extensive cell injury or changes in microglial or astrocytic densities within the hippocampus. In addition, we did not find any difference in blood cytokine levels after PIC injection which excludes the contribution of peripheral inflammation in our model. Together with our in vitro results, one might hypothesize, that even in the absence of cell proliferation, microglial activity is increased by PIC and could be the main source of IL-1β. However, our current data can not exclude the presence of macrophages in the brain tissue. Our results in P14 rats are in line with those of a previous study showing increases in IL-1β in P14 rats after intracerebroventricular (i.c.v.) injection of PIC [33]. TNFα levels were also not changed in this latter study. Further studies are mandatory to determine the exact contribution of the different inflammatory cell types to the production of cytokines in the viral-like inflammation induced by PIC. Moreover, we can not exclude that glial cell activation might occur at later time points.

Intrahippocampal injection of PIC did not change baseline hippocampal excitability in P14 and P75 rats despite leading to increase in IL-1β levels. IL-1β has been consistently described as a factor promoting seizures in both the mature and the immature brain. In adult rats, intrahippocampal injection of IL-1β increases the durations of kainate-induced seizures [34]. In the developing brain, i.c.v. injection of IL-1β leads to pro-convulsant effects in various seizure models [35, 36]. In these former studies, however, IL-1β was directly injected within the brain. By contrast in our model, IL-1β is intrinsically produced thus suggesting that the concentration reached is not sufficient to facilitate ictogenesis.

Interestingly, intrahippocampal injection of PIC facilitated epileptogenesis at both ages. Our results are consistent with the implication that IL-1β has a role in epileptogenesis [37]. This was first suggested by the upregulation of IL-1β in the hippocampi of chronically epileptic mice and in resected hippocampal tissue from patients with temporal lobe epilepsy [38, 39]. The importance of IL-1β in epileptogenesis was further supported by the finding that VX-765, a selective inhibitor of caspase 1, counteracted kindling in adult rats when administered concomitantly with a blockade of IL-1β increase in astrocytes [19]. In agreement with our results, the i.c.v. injection of PIC in P14 rats resulted in an immediate increase in the hippocampal levels of IL-1β without causing any changes in TNFα levels, and was followed by a long-term modification of seizure susceptibility [33], further supporting that IL-1β is a key factor in the facilitation of epileptogenesis by viral-like inflammation.

Minocycline, a semi-synthetic tetracycline antibiotic [40], exerts a variety of biological effects, including proteolysis inhibition, antioxidant effects, and anti-apoptotic and anti-inflammatory activities [23, 41–44]. Minocycline is also well-known to inhibit microglial activation [21, 22, 45]. We first hypothesized that minocycline might modulate the PIC-induced increase in the epileptogenesis through the modulation of inflammation. This assumption was based on previous reports, which have shown that minocycline is able to counteract the long-term increase in brain excitability produced in neonatal rats subjected to an i.c.v. injection of PIC [33]. By decreasing microglial cell activation/proliferation under conditions of status epilepticus induced in juvenile mice by kainate, minocycline limits the kainate-mediated lowering of the seizure threshold [46]. Moreover, the anticonvulsant effects of minocycline have been reported in the amygdala-kindling and the 6-Hz models of adult animals [47, 48]. However, in our model, we did not observe any effect of minocycline on the rapid kindling epileptogenesis and on the pro-epileptic effect of PIC both in P14 and P75 rats. This might be, in part, due to the inability of minocycline to inhibit intrinsic PIC-mediated IL-1β production. Paradoxically, we found a decrease in ADT before and after kindling in P14 rats. This pro-ictogenic effect of minocycline might be associated with some of the paradoxical effects of minocycline described in the developing brain [49] and remains to be studied in details.

## Conclusion

The PIC model allowed us to evaluate the involvement of TLR3 agonists in epileptogenesis. Intrahippocampal injection of PIC induced brain inflammation and in particular increased IL-1β levels as well as facilitated kindling epileptogenesis. PIC produced similar effects in P14 and P75 rats, suggesting that mature and immature brains share common mechanisms in our model. Further research is clearly needed to evaluate the key factors involved in the facilitation of epileptogenesis by viral encephalitis, and more specifically to establish the role of IL-1β.

## Additional file

Additional file 1: Blood cytokine levels 24 h after PIC injection measured by Multiplex cytokine assay. Cytokines profile (IL1β, IL6, IL10, TNFα) in the blood 24 h after intrahippocampal injection (saline i.h (*white bar)* or PIC i.h. (10 μg/rat) (*black bar*) in P14 and in P75 rats. Data are presented as mean ± SEM. We did not find any difference between the groups. (DOCX 9451 kb)

## Abbreviations

AD: Afterdischarge
ADD: Afterdischarge duration
ADT: Afterdischarge threshold
CNS: Central nervous system
DA: Daniel’s
DMEM: Dulbecco’s modified Eagle’s minimum essential medium
EEGs: Electroencephalograms
FBS: Fetal bovine serum
i.p.: Intraperitoneal
IL-10: Interleukin 10
IL-1β: Interleukin 1β
IL-6: Interleukin 6
P13: Postnatal day 13
P74: Postnatal day 74
PIC: Polyinosinic-polycytidylic acid
TLR3: Toll-like receptor 3
TLRs: Toll-like receptors
TMEV: Theiler’s murine encephalomyelitis virus
TNFα: Tumor necrosis factor α
