# Supplementary material for: Pro-epileptogenic effects of viral-like inflammation in both mature and immature brains
Source: J Neuroinflammation. 2016 Dec 12;13:307. doi: 10.1186/s12974-016-0773-6 (PMC5153898; doi:10.1186/s12974-016-0773-6)
Supplement: Additional file 1: — Blood cytokine levels 24 h after PIC injection measured by Multiplex cytokine assay. Cytokines profile (IL1β, IL6, IL10, TNFα) in the blood 24 h after intrahippocampal injection (saline i.h (white bar) or PIC i.h. (10 μg/rat) (black bar) in P14 and in P75 rats. Data are presented as mean ± SEM. We did not find any difference between the groups. (DOCX 9451 kb) [file 12974_2016_773_MOESM1_ESM.docx]

**Supplemental material: Blood level 24 h after hippocampal injection**


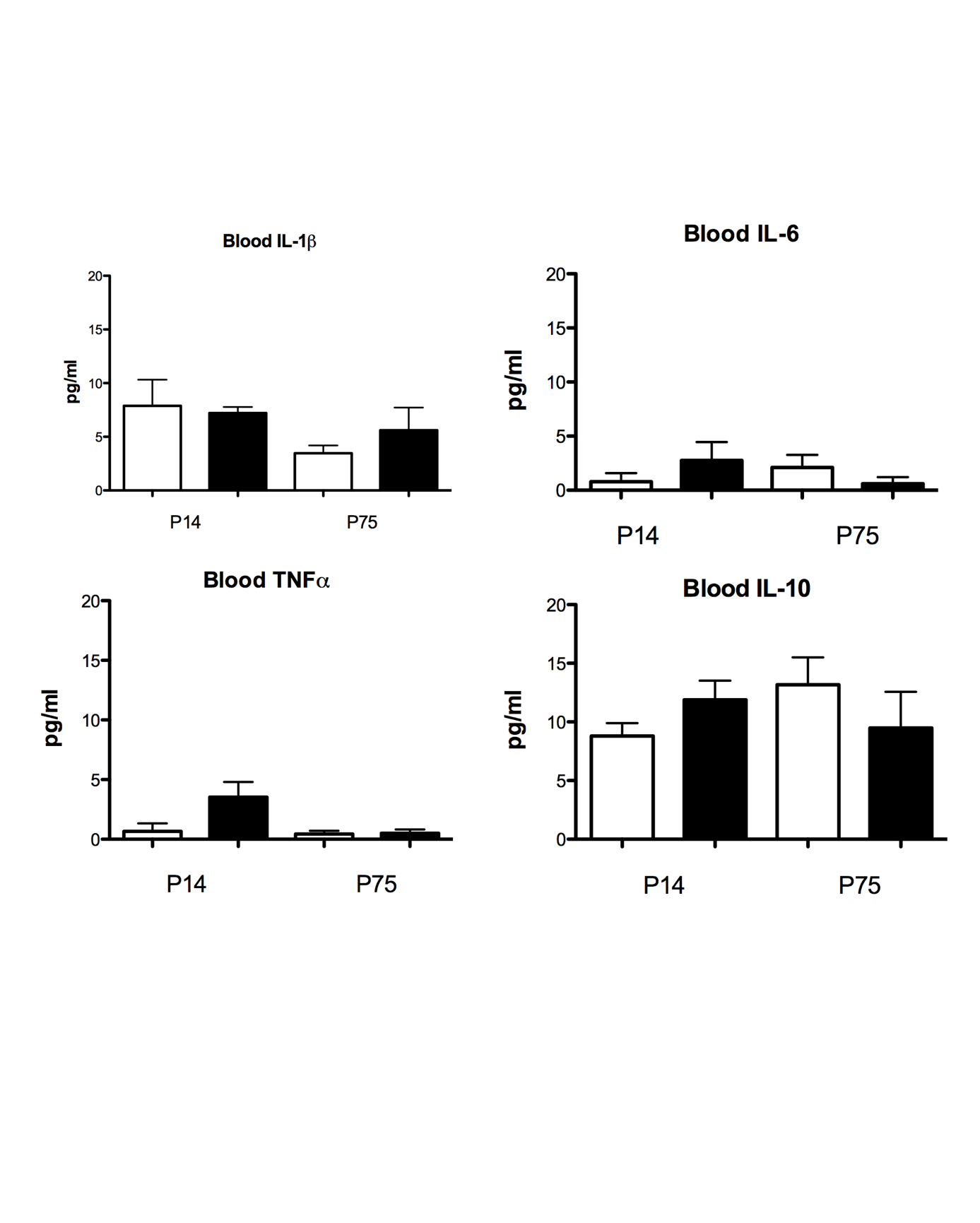


**Blood cytokine levels 24h after PIC injection measured by Multiplex cytokine assay.** Cytokines profile (IL1β, IL6, IL10, TNFα) in the blood 24h after intrahippocampal injection (saline i.h (white bar) or PIC i.h. (10μg/rat) (black bar) in P14 and in P75 rats. Data are presented as mean ± SEM. We did not find any difference between the groups.
